# First Isolation of *Klebsiella pneumoniae* from Septicaemic Piglets in Poland

**DOI:** 10.3390/microorganisms14010256

**Published:** 2026-01-22

**Authors:** Piotr Cybulski, Ines Spiekermeier, Radosław Kondratiuk, Artur Jabłoński, Patryk Tarka, Grzegorz Woźniakowski

**Affiliations:** 1 Goodvalley Agro S.A., Dworcowa 25, 77-320 Przechlewo, Poland; radoslaw.kondratiuk@goodvalley.com; 2 SAN Group Biotech Germany GmbH, Mühlenstrasse 13, 49685 Höltinghausen, Germany; ines.spiekermeier@san-group.com; 3 Department of Pathology and Veterinary Diagnostics, Institute of Veterinary Medicine, Warsaw University of Life Sciences–SGGW, Nowoursynowska 159C, 02-776 Warsaw, Poland; artur_jablonski@sggw.edu.pl; 4 Department of Social Medicine and Public Health, Medical University of Warsaw, Pawińskiego 3a, 02-106 Warsaw, Poland; patryk.tarka@wum.edu.pl; 5 Department of Infectious, Invasive Diseases and Veterinary Administration, Institute of Veterinary Medicine, Faculty of Biological and Veterinary Sciences, Nicolaus Copernicus University in Torun, Lwowska 1, 87-100 Toruń, Poland; grzegorz.wozniakowski@umk.pl

**Keywords:** porcine pathogens, *Klebsiella pneumoniae*, septicaemia, piglets, WGS, Poland

## Abstract

*Klebsiella pneumoniae* is commonly known to cause a vast range of community-acquired or nosocomial infections. The isolation of *K. pneumoniae* has also been noted in diseased food-producing animals, including swine. The main goals of this study were to document clinical manifestation of a septicaemia outbreak in suckling piglets due to *K. pneumoniae* ST25 and provide molecular characterisation of the isolates. For the purpose of this investigation, 13 dead suckling piglets with cyanosis were selected. All the isolates obtained from affected lungs were susceptible to apramycin, ceftiofur, gentamycin, neomycin, and spectinomycin, presented intermediate susceptibility to florfenicol, and were resistant to other tested antibiotics. Histopathological examination of lungs, kidneys, and livers revealed lesions typical of septicaemia. MLST analysis of the isolates demonstrated a complex metabolic profile of the bacteria with genes attributable to the hypervirulent phenotype. To the best of our knowledge, we documented the first outbreak of *K. pneumoniae* septicaemia in suckling piglets reared in Poland.

## 1. Introduction

Among many species of the genus *Klebsiella* within the *Enterobacteriaceae* family, *Klebsiella pneumoniae* is of major clinical importance for both humans and animals. *K. pneumoniae* is a globally distributed, Gram-negative, rod-shaped, non-motile encapsulated bacterium. Widely studied with reference to human infections, *K. pneumoniae* is regarded as an opportunistic pathogen. In recent years, the bacterium has been commonly found resistant to multiple clinically important antibiotics. Having been ranked among the top three causes of multidrug-resistance-related deaths in humans worldwide [1], the bacterium is commonly known to cause a vast range of community-acquired or nosocomial infections [2]. Its pathogenicity primarily relies on four main virulence factors: pili which enable bacterial adherence, capsular polysaccharides facilitating evasion from host immune response, lipopolysaccharides acting as immune activators, and siderophores enhancing iron accumulation from host iron-binding proteins [3,4]. With the prevalence of infections increasing since the 1980s [5], *K. pneumoniae* has been epidemiologically linked to difficult-to-treat pneumonia, urinary tract, wound, and circulatory system infections [6]. As a bacterium thriving in various host niches and ubiquitously found in environmental habitats, including soil, surface and drinking water, sewage, and plants [7], the isolation of *K. pneumoniae* has also been noted in diseased food-producing animals, including swine [8,9]. Nevertheless, the clinical significance of these niches for infection in humans is disputed. Even though available research studies analysing whole-genome sequenced isolates obtained from animal and human sources demonstrated that the transmission between various ecological niches is less common than human-to-human spread of *K. pneumoniae* [10], potential risk of transmission of the bacterium and its dynamics is a major topic in a One Health integrated approach.

To date, few reports identified clinical cases of cow mastitis [11], pneumonic sheep and goats [12], and septicaemic pigs [8,9]. Although *K. pneumoniae* outbreaks causing considerable financial losses were reported in England and Australia in the 2010s [8,9], the prevalence and pathogenicity of the pathogen in swine reared in major pork-producing countries seem to have been largely neglected and consequently under-researched. Apart from these cases, there are no other descriptions demonstrating septicaemia associated with *K. pneumoniae* in pigs. Therefore, the main goals of this study were to define clinical manifestation of a septicaemia outbreak in suckling piglets due to *K. pneumoniae* and provide molecular characterisation of obtained isolates. Our study is the first to provide in-depth data on virulence factors and antimicrobial resistance genes in *K. pneumoniae* isolates obtained from clinical cases in swine reared in Poland.

## 2. Materials and Methods

### 2.1. Study Farm Characteristics

The investigation was carried out in 2025 in a high-performing commercial 5000-sow herd located in Poland (in the West Pomeranian Voivodeship). All the animals were offered dry steam-conditioned (80 °C) barley- and wheat-based pelleted feed pressed into cylindrical pellets. The levels of crude protein, fibre, and fat in the feed offered in a lactation diet were 16.35%, 3.79%, and 5.40%. The animals were reared on a slatted floor under welfare conditions significantly exceeding the legal requirements of Council Directive 2008/120/EC of 18 December 2008 laying down minimum standards for the protection of pigs. Sows were kept in groups during a period starting from the day of service to one week before the expected time of farrowing. During the lactation period the animals were kept in free-farrowing pens. No changes in the management were implemented before the disease outbreak. The farm enforced strict biosecurity rules, including shower in/shower out, same source of feed, own transportation services, fencing, security cameras, and inlets fitted with a protective mesh against insects. No biosecurity violations were noted during the study period.

### 2.2. Health Status of the Farm

Based on regular laboratory monitoring of appropriate biological samples (oral fluid, blood, faeces, tissues) collected by a veterinarian from animals reared at site, the herd was defined as porcine reproductive and respiratory syndrome virus (PRRSV)-negative, *Mesomycoplasma hyopneumoniae*-positive, toxigenic *Pasteurella multocida*-negative, *Actinobacillus pleuropneumoniae*-negative, transmissible gastroenteritis virus (TGEV)-negative, and porcine epidemic diarrhoea virus (PEDV)-negative. All the pregnant gilts and sows were actively immunised prior to the farrow using HIPRASUIS-GLÄSSER, Suiseng Coli/C and Suiseng Diff/A (Laboratorios Hipra S.A., Amer, Spain), Respiporc FLU3, and RESPIPORC FLUpan H1N1 (Ceva Santé Animale, Libourne, France) according to the manufacturers’ recommendations.

### 2.3. Key Performance Indicators

The retrospective production records (i.e., the average number of liveborn piglets per litter, preweaning mortality rates, and the average weaning weight of piglets) were obtained from the farm management using commercial pig production software (Cloudfarms; Cloudfarms AS, Bratislava, Slovakia). Based on real-time input data provided by the farm management, the system ensures valid monitoring data for production performance at the study farm.

### 2.4. Samples

For the purpose of this investigation, a total of 13, 14- to 28-day-old dead suckling piglets which died overnight (4–7 kg body weight) with cyanosis of the extremities and ventrum were selected. To avoid bias, the animals were collected randomly (respecting described discolouration of the skin) from different farrowing rooms. All the sampled individuals developed pulmonary oedema and fine strands of fibrin across the thoracic and abdominal body organ linings. The research material was collected by a veterinarian performing post-mortem examination of the pigs.

Each lung specimen was put into a sterile plastic screw-top specimen jar. Three sets of tissues (lung, liver, kidney) for histopathological examination were collected from randomly selected individuals from the same batch and fixed separately in pre-filled specimen containers with 10% Neutral Buffered Formalin (NBF) solution (HISTOPOT 40 mL; Serosep Ltd., Limerick, Ireland). All the samples were transported overnight to SAN Group Biotech Germany GmbH (Höltinghausen, Germany) ensuring cold chain conditions to be processed using methodology specified in the following descriptions. Ethical review and approval were waived for this study, as the analysed material originated from a routine veterinary diagnostic investigation ordered by the farm owners.

### 2.5. Bacterial Isolation

Aerobic culture samples were individually collected sterile from each lung of every animal. The collected swabs were subjected to the quadrant streaking technique and inoculated onto six distinct agar media: blood agar (ANIVAC^®^, SAN Group Biotech, Höltinghausen, Germany), blood agar supplemented with a nurse strain (*Staphylococcus epidermidis*) (ANIVAC^®^, SAN Group Biotech, Höltinghausen, Germany), cooked blood agar (Thermo Scientific™, Fisher Scientific GmbH, Schwerte, Germany), BROLACIN agar (ANIVAC^®^, SAN Group Biotech, Höltinghausen, Germany), blood agar with neomycin (ANIVAC^®^, SAN Group Biotech, Höltinghausen, Germany), and blood agar with gentamicin (ANIVAC^®^, SAN Group Biotech, Höltinghausen, Germany). The set of plates used for cultivation correspond to a standardised panel for respiratory pathogens of swine at the lab. The inoculated plates were incubated under aerobic conditions at 37 °C for up to 72 h. Macroscopic evaluation of bacterial growth was performed at 24 h intervals. Subcultures were prepared from bacterial cultures to obtain pure cultures for subsequent matrix-assisted laser desorption ionisation–time-of-flight mass spectrometry (MALDI-TOF MS) identification and antimicrobial susceptibility testing.

### 2.6. MALDI-TOF MS Bacterial Identification

On plates where colony growth was observed, bacterial isolates were subsequently verified through MALDI-TOF MS utilising a MALDI Biotyper (Bruker Daltonik GmbH, Bremen, Germany). The MALDI-TOF score value thereby indicated the similarity between a sample’s mass spectrum and a reference spectrum in the database. A score of ≥2.0 signified a high-confidence identification at the species level.

### 2.7. Antimicrobial Susceptibility Testing

Antimicrobial susceptibility testing for *K. pneumoniae* was performed through determination of the Minimal Inhibitory Concentration (MIC) utilising a microtiter plate assay (microtiter plate: DVG food-producing animals MICRONAUT-S Großtiere E1-318-100) and disc diffusion testing. The laboratory procedure adhered strictly to the guidelines set forth by the Clinical and Laboratory Standards Institute (CLSI) document VET01S and the European Society of Clinical Microbiology and Infectious Diseases (EUCAST). The evaluation of the MIC results was performed automatically using the Micronaut6 software system by Bruker (Bruker Corporation, Billerica, MA, USA). The underlying database draws on CLSI, EUCAST, as well as other available literature sources.

### 2.8. Polymerase Chain Reaction (PCR)

For the molecular analysis, several pieces from each lung were combined to create three pooled samples consisting of 5, 4, and 4 lungs collected from all the 13 individuals, respectively (recommended by the PCR kit manufacturers, validation tests have shown that sensitivity is only minimally affected). Extraction of nucleic acids (DNA and RNA) from these pooled lung tissues was conducted using established molecular biology protocols, specifically employing the Kylt^®^ RNA/DNA Purification Kit (SAN Group Biotech Germany GmbH, Höltinghausen, Germany). Subsequent real-time PCR assays were performed to screen for influenza A Virus (Kylt^®^ IVA beta RTU FLI-C 069; SAN Group Biotech Germany GmbH, Höltinghausen, Germany), porcine circovirus type 2 (PCV-2) (Kylt^®^ PCV-2; SAN Group Biotech Germany GmbH, Höltinghausen, Germany), and both European and North American strains of porcine reproductive and respiratory syndrome virus (PRRSV-EU/-NA) (Kylt^®^ PRRSV; SAN Group Biotech Germany GmbH, Höltinghausen, Germany) in all the three pools, following the manufacturers’ recommended procedures.

In the PCR analysis with Kylt^®^ IVA beta RTU and Kylt^®^ PCV-2, an endogenous control (beta actin) is coamplified in each sample. The beta-Actin DNA target gene is ubiquitous in the cells of the host that the sample is derived from. The beta-Actin DNA target gene is co-amplified with every single reaction and allows for evaluation of sufficient sample preparation/DNA extraction and the Real-Time PCR run itself. For the Kylt^®^ PRRSV assay an Internal Control RNA (IC-RNA) is coamplified in each sample. The IC-RNA is added during the RNA preparation and co-purified with each sample. IC-RNA can be detected in the Internal Control channel if the RNA preparation was successful and no RT- or real-time PCR inhibitors are present. All PCR assays used are commercially available and were conducted in full compliance with the manufacturer’s instructions provided on the Kylt^®^ product website. Reference for the PCR assays: SAN Group Biotech Germany GmbH, Höltinghausen, Germany (https://www.kylt.eu/downloads, accessed on 3 December 2025).

### 2.9. Histopathological Examination

The sampled tissues were fixed in 4% neutral buffered formalin to preserve cellular and tissue architecture. Subsequently, the material was embedded in paraffin, sectioned into thin slices, and stained. The prepared slides were examined microscopically, with systematic analysis of tissue architecture, cellular morphology, inflammatory processes, degenerative changes, necrosis, and other pathological alterations. All histopathological investigations were conducted at IVD, Gesellschaft für Innovative Veterinärdiagnostik mbH, Seelze-Letter, Germany.

### 2.10. Bacterial Whole Genome Sequencing (WGS) and Multi-Locus Sequence Typing (MLST) Analysis

Three randomly selected cultures of *K. pneumoniae* obtained during bacterial isolation protocol described above were processed to isolate high-molecular weight DNA for subsequent long-read nanopore sequencing on a MinION device (Oxford Nanopore Technologies, Oxford, UK). An overview of the sequencing throughput is shown in Table 1. The complete genomic sequences of 3 isolates and plasmids have been submitted to GenBank database (https://www.ncbi.nlm.nih.gov/genbank/, accessed on 3 December 2025; publication in progress). All the obtained sequences were typed using the following open-source bioinformatics software tools for multi-locus sequence typing (MLST): Kleborate v2.3.0 (https://github.com/klebgenomics/Kleborate, accessed on 3 December 2025), ResFinder-4.7.2 (https://genepi.food.dtu.dk/resfinder, accessed on 3 December 2025; used settings: treshold identity: 95%, minimum lenght: 80%, database version ResFinder-2.5.1), PlasmidFinder 2.1 (https://cge.food.dtu.dk/services/PlasmidFinder/, accessed on 3 December 2025; used settings: treshold identity: 95%, minimum lenght: 80%), and MLST 2.0 (https://cge.food.dtu.dk/services/MLST/, accessed on 3 December 2025; v2.0.9, database version: 2025-09-15).

### 2.11. Phylogenetic Analysis

The whole-genomic sequence analysis has been conducted on MinION device (Oxford Nanopore Technologies, Oxford, UK) as an outsourcing service. The sequence analysis has been performed in Geneious Prime 2026.0 software (Biomatters, Auckland, New Zealand). Nucleotide alignment has been performed after a sequence similarity search program BLAST 2.17.0 (National Library of Medicine, Bethesda, MD, USA) searching of the most representative *K. pneumoniae* genomes. Alignment has been performed using Muve alignment algorithm. Additionally the plasmid sequences have been aligned using Geneious algorithm.

## 3. Results

### 3.1. Key Performance Indicators

The average number of liveborn piglets per litter in a period of three months preceding the outbreak was 18.73. Concurrently, the preweaning mortality rate was 11.40%, with maternal overlay, runts (including scours), and low birth weight (<600 g) reported as the three main reasons of piglet death, accounting for 40.28% (2800/6952), 33.66% (2340/6952), and 14.37% (999/6952) of dead individuals, respectively. The average weaning weight in this period was 6.25 kg.

The outbreak of *K. pneumoniae* septicaemia was reported in August 2025. Cyanotic suckling piglets in overall good bodily condition from 14 days of age to weaning (4–7 kg body weight) were found dead without pre-diagnosed clinical signs of any disease. In addition to discolouration of the extremities and ventrum, the most frequent gross pathological alterations discovered during postmortem examinations carried out by a veterinarian were pulmonary oedema and fine strands of fibrin across the thoracic and abdominal body organ linings.

The outbreak had sudden onset and was of relatively short duration. Its total timespan was 4 weeks. Total pre-wean mortality during this period was 17.9%. The pre-wean mortality rate attributed to the disease was estimated at 6% with little to no variation between the affected batches. Total individual-litter mortality was from one to three piglets irrespective of the sow parity, with the majority (>70%) of the litters affected.

### 3.2. Intervention

To prevent further spread of the disease between litters reared in different farrowing rooms, the farm management adopted rules set by the Management Changes to Reduce Exposure to Bacteria to Eliminate Losses (McREBEL) system [13]. No groups without the intervention were monitored. Taking into account both identification of the disease in its early stage of spread and widely variable efficacy of clinical interventions reported in the available literature [9], no antibiotic medication was administered. The outbreak was self-limiting. Gradual reduction in mortality rates to the levels preceding the case required a few weeks. All piglets affected within this period were the same age and presented the same symptoms as described above.

### 3.3. Bacterial Isolation and MALDI-TOF MS Bacterial Identification

Of the 13 samples examined, all were positive for bacteria by a microbiological culture. *K. pneumoniae* was successfully isolated from 76.9% (10/13) of the submitted samples (Figure S1). *K. pneumoniae* was isolated from blood agar, blood agar supplemented with a nurse strain, BROLACIN, and cooked blood agar. A total of 15.4% (2/13) of the samples showed an overgrowth by *Proteus* sp. One sample (1/13; 7.7%) was *E. coli*-positive.

### 3.4. Antimicrobial Susceptibility Testing

The results of the antibiotic susceptibility testing for the isolates are shown in Table 2. All the *K. pneumoniae* isolates obtained were susceptible to apramycin, ceftiofur, gentamycin, neomycin, and spectinomycin. A total of 60% (6/10) of the case isolates were susceptible to amoxicillin/clavulanic acid (8/4 µg/mL). All the other presented intermediate susceptibility (16/8 µg/mL). A total of 90% (9/10) of the isolates demonstrated intermediate susceptibility to colistin (0.5 or 1 µg/mL). One isolate was resistant (>2 µg/mL) to the antibiotic. A total of 60% (6/10) of the isolates were susceptible to doxycycline. The rest demonstrated resistance to this antibiotic. A total of 60% (6/10) of the obtained *K. pneumoniae* isolates tested susceptible to gamithromycin (8 µg/mL). All the others were resistant to the antibiotic (>8 µg/mL). A total of 80% (8/10) of the isolates presented intermediate susceptibility to tildipirosin (16 µg/mL). A total of 20% (2/10) were resistant (32 µg/mL) to this antimicrobial. All the case isolates presented intermediate susceptibility to florfenicol (4 µg/mL) and were resistant to the following antibiotics: amoxicillin, ampicillin (>16 µg/mL), erythromycin (>4 µg/mL), lincomycin, oxytetracycline, penicillin (>2 µg/mL), tetracycline (>8 µg/mL), tiamulin (>16 µg/mL), tilmicosin (>16 µg/mL), trimethoprim/sulfamethoxazole (>2/38 µg/mL), tulathromycin (>64 µg/mL), and tylosin.

### 3.5. Polymerase Chain Reaction (PCR)

None of the three pooled lung samples were demonstrated to contain the genetic material of PRRSV, PCV2, or influenza virus type A.

### 3.6. Histopathological Examination

In addition to intravascular presence of rod-shaped bacteria in all types of sampled tissues, histopathological examination revealed the same predominant alterations in all three sets of tissues: mild acute fibrinous and haemorrhagic pneumonia with equally aerated alveoli with distended hyperaemic capillaries, moderate to severe acute hyperaemia of kidney tissue, and severe acute hyperaemia of liver tissue. No hint for preexisting lesions was demonstrated.

### 3.7. Bacterial Whole Genome Sequencing (WGS)

All the samples were subjected to a complete *de novo* bacterial genome assembly workflow and subsequent downstream analyses. Final consensus genomes were used to address coverage, GC content, and completeness using a *Klebsiella*-specific database. Each genome was composed of 1 circular chromosome scaffold and 3 or 4 plasmids. The results of bacterial WGS are shown in Table 3.

### 3.8. Multi-Locus Sequence Typing (MLST) Analysis

Overview of MLST performed on the study isolates is presented in Table 4. All the case isolates were typed as *K. pneumoniae* sequence type 25 (ST25). The analysis revealed complex metabolic profile of the bacteria and several virulence factors potentially associated with the outbreak. All the isolates sequenced in the study harboured the following siderophore systems: several yersiniabactin systems (including *ybt 2*, *ICEKp1*, *YbST 324-1LV*) and one salmochelin system (*iro 3* truncated). Other key virulence genes detected in all the isolates included *rmpA 11*, *rmp3*, *iroB,* and *wzi 72*. One isolate (sample ID 1) harboured aerobactin *iuc3*, *iucA 9*, and *iuC 12*. Table S1 contains detailed data regarding resistance genes and phenotypes. Complete information on genes coding virulence factors is demonstrated in Table S2.

### 3.9. Phylogenetic Analysis

**Figure 1:**
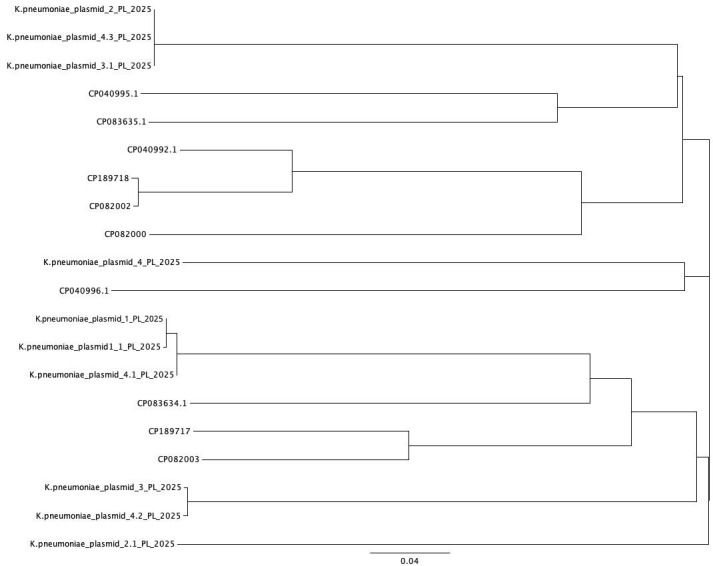
Genetic relationships between *K. pneumoniae* plasmids isolated from pigs in Poland and other isolates originated from clinical infections in human (Geneious Prime, Geneious Alignment).

The conducted phylogenetic analysis of plasmids sequenced from isolates of *K. pneumoniae* revealed their genetic relatedness (Figure 1). Minor genetic diversity has been identified in the case of Plasmid #4, and plasmid 2.1. originated from the same swine holding. The reference plasmids retrieved from GenBank database (provided accession numbers) were significantly divergent from the isolates detected for the first time in Poland. An exception was a plasmid 4_PL from *K. pneumoniae* which was closely related to the CP04966.1 sequence described and published by Sproer et al., 2019, in the USA [14]. The genomes of *K. pneumoniae* from swine revealed differentiated structure (Figure S2), suggesting multiple genetic events which occurred during the adaptation process of these bacteria.

## 4. Discussion

Typical clinical signs of septicaemia include fever, increased heart rate, low blood pressure, severe pain, and lethargy. In our study piglets affected by septicaemia died after a short course of the disease without preceding signs; therefore, prioritising welfare concerns and considerable economic implications of the condition, a definite diagnosis of its primary cause followed by effective intervention are of major importance. The main pathogens traditionally associated with cases of septicaemia in suckling piglets were beta-haemolytic streptococci (predominantly *Streptococcus suis*) [15], enterotoxigenic *Escherichia coli* (ETEC) [16], *Erysipelothrix rhusiopathiae* [17], and *Actinobacillus suis* [18]. The clinical significance of less obvious bacteria seems to have been undervalued. To the best of our knowledge, this is the first documented outbreak of *K. pneumoniae* septicaemia in suckling piglets in Poland. Following its clinical manifestation and laboratory investigation, the case definition was agreed as ‘suckling piglets older than 14 days found dead with lesions corresponding to septicaemia, pure growth of *K. pneumoniae* isolated from lungs, and histopathological lesions of lungs, liver and kidney consistent with septicaemia’.

As a commensal bacterium of the porcine digestive tract, *K. pneumoniae* has been commonly isolated from heathy pig populations [19]. Accordingly, the correct identification of predisposing risk factors compromising immature individuals draws special attention. Contrary to the previously described cases [8,9], pigs sampled in our investigation were reared on slatted floors with no access to bedding materials. Even though it was hypothesised that *K. pneumoniae* mastitis was associated with provision of sawdust or wood shavings in dairy cattle farms and, in a sense, such organic enrichment could have provided a perfect environment for bacterial multiplication and spread [20], no causal link was established between the outbreaks and straw or sawdust offered to swine [8]. Since the vast majority of affected farms in England were outdoor type, the animals could have had unrestricted access to *K. pneumoniae*-contaminated external environmental sources; nevertheless, identification of the same issue in swine reared indoors suggests limited impact of massive bacterial contamination of external origin [9].

Due to its multiple virulence factors, *K. pneumoniae* has been described as a pathogen easily attaching to abiotic surfaces of medical devices that poses a substantial risk of nosocomial infections [21]. Nevertheless, parallel research studies demonstrating the cause-and-effect relationship between the formation of a biofilm in water lines and/or feeding systems and occurrence of the disease at swine farms have not been published to date. Similarly, clinical relevance of asymptomatic carrier animals, including rodents [22], remains highly speculative. According to the available literature, clinically relevant strains of the bacterium were detected in various species of animals, including mice, rats, and shrews [23]; however, strict biosecurity rules established at the study farm effectively prevented the pigs from contact with other animals, their natural habitats, and contaminated vehicles, water, or feed.

Since the first documented outbreak of *K. pneumoniae* septicaemia in England in 2011, all the reported cases in the country have been attributed to a predictable seasonal occurrence–from May to September. Also, the Australian cases were identified during the local summer months. Regardless of potential differences between farm infrastructure, sudden deaths observed during the same season are strongly suggestive of heat stress and/or potentially poorer sanitation levels; nevertheless, the exact role of in-farm environmental factors triggering the outbreaks of *K. pneumoniae* septicaemia requires further spatiotemporal investigation. Taking into consideration good bodily condition of dead piglets reported in all the available studies describing outbreaks of *K. pneumoniae* septicaemia, the influence of variables related to poor colostrum intake and/or nutritional deficiencies can be defined as minor to none.

The age span of affected suckling piglets described in the available case studies varied from one week to weaning. Even though the lower age limit for *K. pneumoniae*-related mortality reported in our investigation was slightly higher (i.e., 14 days), general clinical manifestation of the infection was consonant with those demonstrated in previous research. In all the officially reported cases, affected individuals in good bodily condition were found dead and cyanosed. In our study, total pre-wean mortality rate attributed to *K. pneumoniae* septicaemia at 6% was broadly within the range reported in English herds (from 0.8 to 15.8%); however, it was still markedly lower than those in Australian farms–up to 60% in Victorian, and up to 100% in Queensland locations. It is noteworthy that animals reared in the latter facilities tested positive for encephalomyocarditis virus (EMCV), which could have profoundly influenced clinical manifestation of the disease. Similarly to the English report, involvement of concurrent infections was systematically excluded in our investigation using a set of microbiological, molecular, and histopathological testing.

MLST analysis revealed that all three *K. pneumoniae* isolates obtained from septicaemic piglets sampled in our investigation were sequence type 25 (ST25). The same sequence type was already associated with the outbreaks in England, where all 25 case isolates obtained from septicaemic individuals (representing all 15 reported outbreaks) were identified as *K. pneumoniae* ST25 [9]. Also, two out of four septicaemia outbreaks in suckling piglets reported in Victoria, Australia were associated with the same sequence type of the bacterium.

Based on increased expression fimbriae, siderophores, and hypermycoviscosity, *K. pneumoniae* strains can be categorised as classical or hypervirulent isolates. With the exception of one English piece of research, there are no other peer-reviewed scientific works on virulence factors of *K. pneumoniae* isolates obtained from septicaemic pigs. The analysis conducted on 13 ST25 isolates demonstrated presence of *rmpA* gene, associated with upregulated capsule expression and the hypermucoviscous phenotype [24]. The same gene was found in all three isolates analysed in our study. In addition to *rmpA*, all the ST25 isolates displayed other hypervirulence characteristics, including the presence of salmochelin (*iro3*), yersiniabactin (*ybt2*), and *iroB*.

Direct comparison of antibiotic susceptibility between available case isolates is severely limited by the usage of different panels of antimicrobials. Also, CLSI does not provide interpretive criteria for susceptibility testing of the bacterium. As expected, all the *K. pneumoniae* isolates obtained in our study confirmed their innate resistance to ampicillin. Contrary to the English outputs, all the tested Polish case isolates were sensitive to spectinomycin and apramycin. The same findings regarding the latter antibiotic were reported in the Australian piece of research. Isolates obtained during our investigation demonstrated a resistance pattern to sulphonamides and trimethoprim which also corroborates the Australian report where some of the isolates were found to be resistant against these antibiotics. The same study demonstrated partial resistance to neomycin, which is contrary to our results. Overall, the clinical significance of these results may be severely limited since in vitro findings do not necessarily translate to in vivo efficacy.

Commercial vaccines against *K. pneumoniae*-associated diseases are not available. Also, data on basic effectiveness of autogenous bacterins containing *K. pneumoniae* have not been published to date. Although antimicrobial susceptibility testing showed that the isolates obtained in our investigation were sensitive to various commercially available antibiotics commonly used in swine, no antimicrobial-based clinical intervention was executed at the farm. The implementation of the McREBEL system led to self-limitation of the outbreak; still, it was technically impossible to provide a proper baseline for comparison using a control group. Consequently, evaluation of the overall impact exerted by these measures remains entirely hypothetical, and no casual inference can be drawn. In contrast to our report, available case studies documented the administration of various treatments, including parenteral medication of cohorts in affected litters, medication offered via drinking water, or medicated creep feed. Nevertheless, clinical efficacy of these interventions cannot be systematically evaluated without implementation of control groups.

The only available piece of research demonstrating histopathological alterations in pigs experimentally infected with *K. pneumoniae* reveals alveolar capillary congestion and dilatation, activation of inflammatory cells, and septal thickening of the tissue following the intranasal administration [25]. Reports on the virulence of the bacterium on porcine tissues in septicaemic individuals have not been published to date. In spite of the fact that the dead suckling piglets autopsied during our investigation showed gross lesions largely consistent with those seen in other septicaemic infections typical of that age, pure growth of the bacterium in a number of samples obtained from a visceral site of affected individuals was obtained. Also, intravascular presence of bacteria morphologically corresponding to *K. pneumoniae* was proven during histopathological examination of clinical samples representing two other vital organs obtained from the same piglets, i.e., the liver and kidneys.

Detailed description of clinical presentation (i.e., good body condition of the animals and spread of the affected individuals across different litters) directly exclude the influence of problems related to low colostrum intake or nutritional deficiencies. Also, other non-infectious causes of preweaning mortality can be automatically excluded–the effects of hypoxia, slurry gas poisoning, or electric shock would not be spread across individuals reared in different pens. Since no concurrent viral or bacterial diseases were identified, and histopathological examination of visceral sites demonstrated no hint of pre-existing alterations, all these features are strongly suggestive that *K. pneumoniae* was an aetiological agent of the septicaemia outbreak. Consequently, our study is the first to summarise the main histopathological lesions found in *K. pneumoniae*-related septicaemia in piglets.

## 5. Conclusions

Our research highlights the importance of less obvious porcine pathogens triggering septicaemia in suckling piglets. To the best of our knowledge, this is the first documented outbreak of *K. pneumoniae septicaemia* in suckling piglets in Poland. All the case isolates were typed as *K. pneumoniae* sequence type 25 (ST25). The analysis revealed the complex metabolic profile of the bacteria with genes attributable to the hypervirulent phenotype: *rmpA, iro3*, *ybt2*, and *iroB*. Taking into account a One Health approach, the emergence of *K. pneumoniae* ST25 in swine reared in Poland represents a health concern; therefore, potential risk factors associated with outbreaks and spread of the disease deserve further spatiotemporal investigation.

## Figures and Tables

**Table 1:** Overview of sequencing throughput for all samples.

Sample ID	DNA Concentration	Reads	N_50_ (bp) *	Output (Bases)
1	296 ng/μL	648,469.0	7995.0	4,463,124,404.0
2	329 ng/μL	356,214.0	8199.0	2,537,927,730.0
3	326 ng/μL	418,811.0	8086.0	2,876,724,730.0

* As the minimal read size contributing to 50% of the total sequenced output.

**Table 2:** Overall determination of antimicrobial susceptibility of *Klebsiella pneumoniae* isolates obtained from septicaemic suckling piglets from 14 days to weaning.

		KP-1	KP-2	KP-3	KP-4	KP-5	KP-6	KP-7	KP-8	KP-9	KP-10
	Amoxicillin/Clavulanic Acid	16/8	16/8	8/4	8/4	8/4	8/4	8/4	16/8	16/8	8/4
Minimal Inhibitory Concentrations	Ampicillin	>16	>16	>16	>16	>16	>16	>16	>16	>16	>16
	Ceftiofur	0.5	0.5	0.5	0.5	1	0.5	0.5	0.5	0.25	1
	Colistin	≤0.5	1	≤0.5	≤0.5	≤0.5	≤0.5	1	1	≤0.5	>2
	Enrofloxacin	1	0.0625	0.03125	0.03125	0.03125	0.03125	0.03125	0.03125	0.03125	0.03125
	Erythromycin	>4	>4	>4	>4	>4	>4	>4	>4	>4	>4
	Florfenicol	4	4	4	4	4	4	4	4	4	4
	Gamithromycin	>8	>8	8	8	8	>8	8	>8	8	8
	Gentamycin	0.25	0.25	0.25	0.25	0.25	0.25	0.25	0.25	0.25	0.25
	Penicillin	>2	>2	>2	>2	>2	>2	>2	>2	>2	>2
	Tetracyclin	>8	>8	>8	>8	>8	>8	>8	>8	>8	>8
	Tiamulin	>16	>16	>16	>16	>16	>16	>16	>16	>16	>16
	Tildipirosin	16	16	32	16	16	16	16	32	16	16
	Tilmicosin	>16	>16	>16	>16	>16	>16	>16	>16	>16	>16
	Trimethoprim/Sulfamethoxazole	>2/38	>2/38	>2/38	>2/38	>2/38	>2/38	>2/38	>2/38	>2/38	>2/38
	Tulathromycin	>64	>64	>64	>64	>64	>64	>64	>64	>64	>64
Disc Diffusion Test	Amoxicillin	R	R	R	R	R	R	R	R	R	R
	Apramycin	S	S	S	S	S	S	S	S	S	S
	Doxycycline	R	S	S	S	R	S	S	S	R	R
	Lincomycin	R	R	R	R	R	R	R	R	R	R
	Neomycin	S	S	S	S	S	S	S	S	S	S
	Oxytetracyclin	R	R	R	R	R	R	R	R	R	R
	Spectinomycin	S	S	S	S	S	S	S	S	S	S
	Tylosin	R	R	R	R	R	R	R	R	R	R

KP-*n*—*K. pneumoniae* isolate number; Minimal Inhibitory Concentrations (MIC) values reported in µg/mL: green—susceptible; yellow—intermediate susceptible; red—resistant; Disc Diffusion Test (DDT): S—susceptible; R—resistant, green—susceptible; red—resistant.

**Table 3:** Overview of consensus genome quality control.

Sample ID	Coverage	GC Content	Completeness *	Genome Size (bp)	Scaffolds
1	679x	56.85%	99.99%	5,906,606	5
2	393x	57.13%	99.99%	5,686,917	4
3	445x	57.12%	99.99%	5,718,523	4

* Based on 1359 marker genes from 29 genomes.

**Table 4:** Overview of multi-locus sequence typing (MLST) for *K. pneumoniae* isolates obtained from septicaemic suckling piglets from 14 days to weaning.

Sample ID	1	2	3
Quality Module	Passed	Passed	Passed
Genotype-AMR	aac(3)-IV, aph(3′’)-Ib, aph(3′)-Ia, aph(6)-Id, blaSHV-81, blaTEM-1B, dfrA1, dfrA5, fosA6, lnu(G), mph(E), msr(E), OqxA, OqxA, OqxB, OqxB, sul2, sul2, tet(A), tet(D)	aph(3′’)-Ib, aph(6)-Id, blaSHV-81, blaTEM-1B, dfrA5, fosA6, OqxA, OqxA, OqxB, OqxB, sul2	aph(3′’)-Ib, aph(6)-Id, blaSHV-81, blaTEM-1B, dfrA5, fosA6, OqxA, OqxA, OqxB, OqxB, sul2, tet(A)
Predicted Phenotype	gentamicin, tobramycin, streptomycin, kanamycin, ampicillin, trimethoprim, fosfomycin, lincomycin, erythromycin, azithromycin, uknown[OqxA_1_EU370913], unknown[OqxB_1_EU370913], sulfisoxazole, tetracycline	streptomycin, kanamycin, ampicillin, trimethoprim, fosfomycin, uknown [OqxA_1_EU370913], unknown[OqxB_1_EU370913], sulfisoxazole	streptomycin, kanamycin, ampicillin, trimethoprim, fosfomycin, unknown[OqxA_1_EU370913], unknown[OqxB_1_EU370913], sulfisoxazole, tetracycline
Plasmid	IncFIB(K), IncI1-I(Alpha), IncX5	IncI1-I(Alpha), IncFII	IncI1-I(Alpha), IncFII
Sequence Type (ST)	25	25	25
Genome Length (bp)	5,906,606	5,686,917	5,718,523
N50 value	5,384,397	5,385,583	5,386,774

## Data Availability

The original contributions presented in this study are included in the article/Supplementary Material. Further inquiries can be directed to the corresponding author.

## Supplementary Materials

The following supporting information can be downloaded at https://www.mdpi.com/article/10.3390/microorganisms14010256/s1, Figure S1: Colony morphology of *Klebsiella pneumoniae* in pure culture on blood-agar medium isolated from a lung sample collected from a septicaemic 3-week-old piglet; Figure S2: Genomic alignment *Klebsiella pneumoniae* whole genomes isolated from pigs in Poland and other isolates originated from clinical infections in human (Geneious Prime; Muve genome alignment); Table S1: Overview of resistance genes and phenotypes for *K. pneumoniae* isolates obtained from septicaemic suckling piglets from 14 days to weaning; Table S2: Overview of virulence factors for *K. pneumoniae* isolates obtained from septicaemic suckling piglets from 14 days to weaning.

## Abbreviations

The following abbreviations are used in this manuscript:

bp	base pair
DDT	Disc Diffusion Test
DNA	Deoxyribonucleic acid
EMCV	encephalomyocarditis virus
ETEC	enterotoxigenic Escherichia coli
MALDI-TOF MS	matrix-assisted laser desorption ionisation-time-of-flight mass spectrometry
McREBEL	Management Changes to Reduce Exposure to Bacteria to Eliminate Losses
MIC	Minimal Inhibitory Concentration
MLST	multi-locus sequence typing
NBF	neutral buffered formalin
PCR	polymerase chain reaction
PCV2	porcine circovirus type 2
PEDV	porcine epidemic diarrhoea virus
PRRSV	porcine reproductive and respiratory syndrome virus
TGEV	transmissible gastroenteritis virus
WGS	Whole Genome Sequencing
