# Supplementary material for: First Isolation of Klebsiella pneumoniae from Septicaemic Piglets in Poland
Source: Microorganisms. 2026 Jan 22;14(1):256. doi: 10.3390/microorganisms14010256 (PMC12844330; doi:10.3390/microorganisms14010256)
Supplement: Supplementary file 1 [file microorganisms-14-00256-s001.zip › microorganisms-4053403-supplementary.pdf]

microorganisms

MDPI

Table S1. Overview of resistance genes and phenotypes for *K. pneumoniae* isolates obtained from septicaemic suckling piglets from 14 days to weaning.

Sample ID	Resistance gene	Identity	Alignment Length/Gene Length	Coverage	Position in reference	Contig	Plasmid type	Position in contig	Phenotype	Accession no.
1	aph(6)-Id	100.00	823/837	98.3	1..823	plasmid003_cluster_0 0-114947	IncI1-I(Alpha)	74929..75751	Streptomycin	M28829
	aph(3'')-Ib	100.00	804/804	100.0	1..804	plasmid003_cluster_0 0-114947	IncI1-I(Alpha)	75751..76554	Streptomycin	AF321551
	aph(3')-Ia	100.00	816/816	100.0	1..816	plasmid004_cluster_0 0-71827	IncX5	10788..11603	Kanamycin, Neomycin, Kanamycin, Lividomycin, Paromomycin, Ribostamycin, Unknown Aminoglycoside, Kanamycin, Neomycin	X62115
	aac(3)-IV	100.00	777/777	100.0	1..777	plasmid004_cluster_0 0-71827	IncX5	16050..16826	Gentamicin, Tobramycin	DQ241380
	blaTEM-1B	100.00	861/861	100.0	1..861	plasmid003_cl	IncI1-I(Alpha)	80590..81450	Amoxicillin, Ampicillin, Cephalothin, Piperacillin, Ticarcillin	AY458016

					uster_0 0-114947				
blaSHV-81	99.77	861/861	100.0	1..861	genome_cluster_0 0-5384397	-	2772699..2773559	Amoxicillin, Ampicillin, Cephalothin, Piperacillin, Ticarcillin	AM176556
fosA6	99.05	420/420	100.0	1..420	genome_cluster_0 0-5384397	-	4670995..4671414	Fosfomycin	KU254579
msr(E)	100.00	1476/1476	100.0	1..1476	plasmid004_cluster_0 0-71827	IncX5	13625..15100	Erythromycin, Azithromycin, Quinupristin, Pristinamycin IA, Virginiamycin S	FR751518
mph(E)	100.00	885/885	100.0	1..885	plasmid004_cluster_0 0-71827	IncX5	12685..13569	Erythromycin	DQ839391
lnu(G)	100.00	804/804	100.0	1..804	plasmid004_cluster_0 0-71827	IncX5	25500..26303	Lincomycin	KX470419
OqxB	98.70	3153/3153	100.0	1..3153	genome_cluster_0 0-5384397	-	1169559..1172711	Chloramphenicol, Nalidixic acid, Ciprofloxacin, Trimethoprim	EU370913
OqxA	99.57	1176/1176	100.0	1..1176	genome_clus	-	1172735..1173910	Chloramphenicol, Nalidixic acid, Ciprofloxacin, Trimethoprim	EU370913

					ter_0 0-5384397				
sul2	100.00	816/816	100.0	1..816	plasmid003_cluster_0 0-114947	IncI1-I(Alpha)	76615..77430	Sulfamethoxazole	HQ840942
sul2	100.00	816/816	100.0	1..816	plasmid004_cluster_0 0-71827	IncX5	30922..31737	Sulfamethoxazole	AY034138
tet(A)	100.00	1200/1200	100.0	1..1200	plasmid003_cluster_0 0-114947	IncI1-I(Alpha)	64884..66083	Doxycycline, Tetracycline	AJ517790
tet(D)	100.00	1185/1185	100.0	1..1185	plasmid004_cluster_0 0-71827	IncX5	19382..20566	Doxycycline, Tetracycline	AF467077
dfrA5	100.00	474/474	100.0	1..474	plasmid003_cluster_0 0-114947	IncI1-I(Alpha)	82891..83364	Trimethoprim	X12868
dfrA1	100.00	474/474	100.0	1..474	plasmid004_cluster_0 0-71827	IncX5	35589..36062	Trimethoprim	X00926
-	-	-	-	-	plasmid001_cl	IncFIB(K)	-	-	-

https://doi.org/10.3390/microorganisms14010256

						uster_0 0-225313				
	aph(6)-Id	100.00	823/837	98.3	1..823	plasmid002_cluster_0 0-115032	IncI1-I(Alpha)	104778..105600	Streptomycin	M28829
	aph(3'')-Ib	100.00	804/804	100.0	1..804	plasmid002_cluster_0 0-115032	IncI1-I(Alpha)	103975..104778	Streptomycin	AF321551
	blaTEM-1B	100.00	861/861	100.0	1..861	plasmid002_cluster_0 0-115032	IncI1-I(Alpha)	99079..99939	Amoxicillin, Ampicillin, Cephalothin, Piperacillin, Ticarcillin	AY458016
2	blaSHV-81	99.77	861/861	100.0	1..861	genome_cluster_0 0-5385597	-	2773889..2774749	Amoxicillin, Ampicillin, Cephalothin, Piperacillin, Ticarcillin	AM176556
	fosA6	99.05	420/420	100.0	1..420	genome_cluster_0 0-5385597	-	4672182..4672601	Fosfomycin	KU254579
	OqxB	98.70	3153/3153	100.0	1..3153	genome_cluster_0 0-5385597	-	1169558..1172710	Chloramphenicol, Nalidixic acid, Ciprofloxacin, Trimethoprim	EU370913
	OqxA	99.57	1176/1176	100.0	1..1176	genome_clus	-	1172734..1173909	Chloramphenicol, Nalidixic acid, Ciprofloxacin, Trimethoprim	EU370913

						ter_0 0-5385597				
	sul2	100.00	816/816	100.0	1..816	plasmid002_cluster_0 0-115032	IncI1-I(Alpha)	103099..103914	Sulfamethoxazole	HQ840942
	dfrA5	100.00	474/474	100.0	1..474	plasmid002_cluster_0 0-115032	IncI1-I(Alpha)	97165..97638	Trimethoprim	X12868
	-	-	-	-	-	plasmid001_cluster_0 0-225313	IncFII	-	-	-
3	aph(6)-Id	100.00	823/837	98.3	1..823	plasmid002_cluster_0 0-114946	IncI1-I(Alpha)	97912..98734	Streptomycin	M28829
	aph(3'')-Ib	100.00	804/804	100.0	1..804	plasmid002_cluster_0 0-114946	IncI1-I(Alpha)	98734..99537	Streptomycin	AF321551
	blaTEM-1B	100.00	861/861	100.0	1..861	plasmid002_cluster_0 0-114946	IncI1-I(Alpha)	103573..104433	Amoxicillin, Ampicillin, Cephalothin, Piperacillin, Ticarcillin	AY458016
	blaSHV-81	99.77	861/861	100.0	1..861	genome_clus	-	2773898..2774758	Amoxicillin, Ampicillin, Cephalothin, Piperacillin, Ticarcillin	AM176556

https://doi.org/10.3390/microorganisms14010256

					ter_0 0-5386797				
fosA6	99.05	420/420	100.0	1..420	genome_cluster_0 0-5386797	-	4673394..4673813	Fosfomycin	KU254579
OqxB	98.70	3153/3153	100.0	1..3153	genome_cluster_0 0-5386797	-	1169559..1172711	Chloramphenicol, Nalidixic acid, Ciprofloxacin, Trimethoprim	EU370913
OqxA	99.57	1176/1176	100.0	1..1176	genome_cluster_0 0-5386797	-	1172735..1173910	Chloramphenicol, Nalidixic acid, Ciprofloxacin, Trimethoprim	EU370913
sul2	100.00	816/816	100.0	1..816	plasmid002_cluster_0 0-114946	IncI1-I(Alpha)	99598..100413	Sulfamethoxazole	HQ840942
tet(A)	100.00	1200/1200	100.0	1..1200	plasmid002_cluster_0 0-114946	IncI1-I(Alpha)	87867..89066	Doxycycline, Tetracycline	AJ517790
dfrA5	100.00	474/474	100.0	1..474	plasmid002_cluster_0 0-114946	IncI1-I(Alpha)	105874..106347	Trimethoprim	X12868
-	-	-	-	-	plasmid001_cl	IncFII	-	-	-

	uster_0 0-225313

Table S2. Overview of virulence factors for *K. pneumoniae* isolates obtained from septicaemic suckling piglets from 14 days to weaning.

**Isolate ID**	**1**	**2**	**3**
species	Klebsiella pneumoniae	Klebsiella pneumoniae	Klebsiella pneumoniae
ST	ST25	ST25	ST25
virulence_score	4	1	1
resistance_score	0	0	0
num_resistance_classes	6	4	5
num_resistance_genes	14	5	6
Yersiniabactin	ybt 2; ICEKp1	ybt 2; ICEKp1	ybt 2; ICEKp1
YbST	324-1LV	324-1LV	324-1LV
Colibactin	-	-	-
CbST	0	0	0
Aerobactin	iuc 3	-	-
AbST	25	0	0
Salmochelin	iro 3 (truncated)	iro 3 (truncated)	iro 3 (truncated)
SmST	7	7	7
RmpADC	rmp 3; ICEKp1	rmp 3; ICEKp1	rmp 3; ICEKp1
RmST	32-1LV	32-1LV	32-1LV
rmpA2	-	-	-
wzi	wzi72	wzi72	wzi72
K_locus	KL2	KL2	KL2
K_type	K2	K2	K2
K_locus_problems	none	none	none

K_locus_confidence	Very high	Very high	Very high
K_locus_identity	98.41%	98.41%	98.41%
K_locus_missing_genes			
O_locus	O1/O2v2	O1/O2v2	O1/O2v2
O_type	O2afg	O2afg	O2afg
O_locus_problems	none	none	none
O_locus_confidence	Very high	Very high	Very high
O_locus_identity	99.19%	99.19%	99.19%
O_locus_missing_genes			
AGly_acquired	aac(3)-IV;aph(3')-Ia;strA.v1^;strB.v1?	strA.v1^;strB.v1?	strA.v1^;strB.v1?
Col_acquired	-	-	-
Fcyn_acquired	-	-	-
Flq_acquired	-	-	-
Gly_acquired	-	-	-
MLS_acquired	lnuG;mphE.v2;msrE	-	-
Phe_acquired	-	-	-
Rif_acquired	-	-	-
Sul_acquired	sul2;sul2*	sul2*	sul2*
Tet_acquired	tet(A).v1;tet(D)	-	tet(A).v1
Tgc_acquired	-	-	-
Tmt_acquired	dfrA1.v1;dfrA5	dfrA5	dfrA5
Bla_acquired	TEM-1D.v1^	TEM-1D.v1^	TEM-1D.v1^
Bla_inhR_acquired	-	-	-
Bla_ESBL_acquired	-	-	-
Bla_ESBL_inhR_acquired	-	-	-
Bla_Carb_acquired	-	-	-
Bla_chr	SHV-11.v1^	SHV-11.v1^	SHV-11.v1^
SHV_mutations	35Q	35Q	35Q
Omp_mutations	-	-	-

https://doi.org/10.3390/microorganisms14010256

Col_mutations	-	-	-
Flq_mutations	-	-	-
truncated_resistance_hits	-	-	-
spurious_resistance_hits	-	tet(A).v1?-0%;tet(A).v1?-52%	-
Chr_ST	ST25	ST25	ST25
gapA	2	2	2
infB	1	1	1
mdh	1	1	1
pgi	1	1	1
phoE	10	10	10
rpoB	4	4	4
tonB	13	13	13
ybtS	8	8	8
ybtX	7	7	7
ybtQ	9	9	9
ybtP	6	6	6
ybtA	5	5	5
irp2	13	13	13
irp1	36*	36*	36*
ybtU	6	6	6
ybtT	7	7	7
ybtE	7	7	7
fyuA	6	6	6
clbA	-	-	-
clbB	-	-	-
clbC	-	-	-
clbD	-	-	-
clbE	-	-	-
clbF	-	-	-

https://doi.org/10.3390/microorganisms14010256

clbG	-	-	-
clbH	-	-	-
clbI	-	-	-
clbL	-	-	-
clbM	-	-	-
clbN	-	-	-
clbO	-	-	-
clbP	-	-	-
clbQ	-	-	-
iucA	9	-	-
iucB	7	-	-
iucC	12	-	-
iucD	8	-	-
iutA	17	-	-
iroB	6	6	6
iroC	19-4%	19-4%	19-4%
iroD	10	10	10
iroN	5	5	5
rmpA	11	11	11
rmpD	5*	5*	5*
rmpC	6	6	6

https://doi.org/10.3390/microorganisms14010256
